# The Half-Yearly Abstract of the Medical Sciences

**Published:** 1847-08

**Authors:** 


ARTICLE XIV.

*The Half-Yearly Abstract of the Medical Sciences, &c., &c.* Edited by W. H. RANKING, M.D., Cantab., Physician to Suffolk General Hospital. Assisted by W. A. GUY, M.D., GEORGE DAY, M.D., HENRY ANCEL, M.D., and W. KIRKES, M.D. No. 5, from January to July, 1847. Lindsay & Blakiston, Philadelphia, &c. (In Exchange.)

This deservedly popular work fully sustains its former reputation in this number. It is a full and interesting abstract of what is new in the different departments of our science. As a work of reference it is exceedingly convenient and valuable on account of the admirable arrangement of its contents, and the clear and full accounts given of the many improvements and discoveries which are being made during each succeeding six months. This number contains 364 pages, finely printed and well executed. It is afforded at the low price of $1 50 per annum. E.

